# Sustainable Extraction of Bioactive Compounds from *Annona muricata L.* Leaves by Deep Eutectic Solvents (DESs)

**DOI:** 10.1021/acsomega.5c01232

**Published:** 2025-04-02

**Authors:** Lais F. Oton, Paulo R. V. Ribeiro, Edy S. de Brito, Rílvia S. de Santiago-Aguiar

**Affiliations:** † Chemical Engineering Department, Federal University of Ceará, Pici Campus, Bloco 731B, Fortaleza, CE 60440-900, Brazil; ‡ Embrapa Tropical Agroindustry, Rua Dra Sara Mesquita 2270, Planalto do Pici, CEP, Fortaleza, CE 60511-110, Brazil

## Abstract

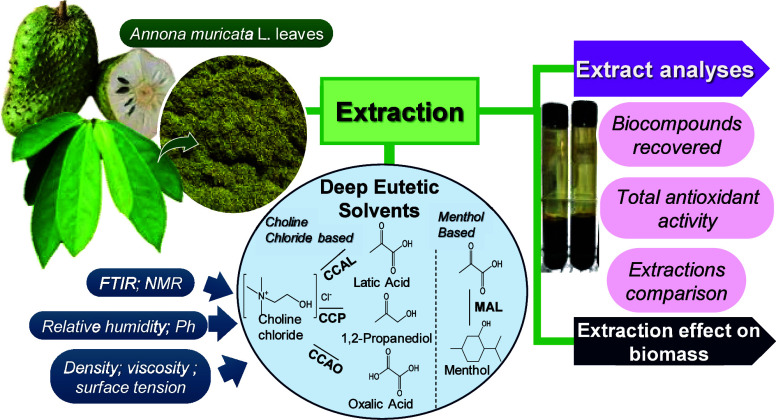

Soursop (*Annona muricata L.*) leaves are rich in bioactive compounds with promising pharmacological and food applications. Deep eutectic solvents (DESs), a class of green and tunable solvents, offer an efficient and sustainable alternative to their extraction. This study investigates the use of various DES formulations for extracting bioactive compounds from soursop leaves under optimized conditions, considering the temperature, solvent-to-biomass ratio, and extraction time in a solid–liquid system. Conventional techniques, such as magnetic stirring and ultrasonic bath extraction, were also evaluated for comparison. DESs were prepared using choline chloride and menthol as hydrogen bond acceptors (HBAs) combined with lactic acid, oxalic acid, and 1,2-propanediol as hydrogen bond donors (HBDs). The optimal extraction conditions were determined at 50 °C with a biomass-to-solvent ratio of 1:10 (m/v). Solvent performance and interactions with biomass were analyzed using NMR, FTIR, density, viscosity, pH, and total humidity assessments. Compared to water and ethanol, DESs exhibited superior efficiency and stability, enhancing cell wall disruption and improving extraction yields. Among the tested solvents, acidic DES (CCAO) demonstrated the highest extraction efficiency despite its high viscosity and density. These findings pave the way for future applications in the pharmaceutical and food industries, reinforcing DESs as a promising environmentally friendly alternative for the extraction of high-value bioactive compounds from plant biomass.

## 1 Introduction

Nature offers a vast array of resources capable of aiding in the treatment of various diseases and illnesses. Traditional knowledge can serve as a valuable guide in identifying raw materials with curative potential. In this context, the soursop tree (*Annona muricata L.*), a member of the Annonaceae family and characteristic of tropical climates, has been widely used in indigenous and alternative medicine. It is particularly valued for its potential to treat conditions such as insomnia, parasitic infections, neuralgia, rheumatism, and cancer.^1−7^

Phytochemical analyses of soursop roots, leaves, and fruits have revealed the presence of numerous bioactive compounds, including flavonoids, coumarins, alkaloids, cardiac glycosides, lactones, acetogenins, and phenols.^2,8−10^ Experimental in vitro and in vivo studies have demonstrated that small doses of soursop leaf extracts exhibit selective toxicity. These bioactive compounds specifically disrupt the metabolism of inflamed cells or pathogens without harming healthy cells in the human body.^3,4,11^

Until now, most methods applied for the extracting and purifying of biochemicals from soursop leaves have relied on conventional procedures, such as pressurization and mechanical agitation, in the presence of volatile solvents such as methanol and chloroform. These methods are often chosen due to their low cost and the ease of separation after the extraction step.^2,12−16^

However, due to the high toxicity of these solvents, the additional steps required for product purification at the end of the extraction process, and the energy costs involved in the applied unit operations, it is necessary to search for green procedures that are applicable on a large scale while maintaining or even improving the quality of the final product.^17−19^ An alternative would be the application of solid–liquid extraction combined with nontoxic solvents that actively participate in the extraction process through a dual mechanism: penetrating the plant cell wall and facilitating the diffusion and chemical stability of the target extractives within the system.

Among the solvents that can be adapted to these functions, DESs stand out. These solvents are prepared through the electrostatic interaction between an HBD component, such as sugars, alcohols, and carboxylic acids, and an HBA component, typically a quaternary ammonium salt.^20−22^ According to the literature, when these components are subjected to a specific temperature and ideal composition, they form a eutectic mixture with a lower melting point. This characteristic ensures that the mixture remains in liquid form even after returning to room temperature.^16,20,23,24^

Studies regarding the extraction of bioactive compounds from biomass in the presence of DESs indicate that the physicochemical characteristics of DESs, such as acidity, polarity, and affinity with water, strongly contribute to the selectivity and efficiency of the extraction, surpassing results obtained with conventional solvents under the same operating conditions.^17,25^ Moreover, DESs have been increasingly recognized due to their high biocompatibility particularly with polyphenols, biodegradability, association with green chemistry, and low production cost combined with the possibility of recycling.^26,27^ Despite these advantages, issues such as mass transfer limitations caused by the high viscosity of DESs, mixture stability, and the potential degradation of extractives remain subjects of analysis and investigation.^17,28^

Thus, this study was developed to evaluate the application of different types of DES in the extraction efficiency of biochemicals from soursop leaves under optimal operating conditions, including the temperature, solvent-to-biomass ratio, and extraction time in a solid–liquid system. Furthermore, this work aims to compare conventional extraction techniques, such as mechanical agitation and ultrasonic baths, to identify the most efficient method for extracting bioactive compounds in the presence of DESs.

## 2 Experimental Section

### 2.1 Vegetal Material

The leaves were obtained from the reuse of soursop tree pruning residues in the Chapada do Apodi region, Limoeiro do Norte-CE, Brazil (latitude: 5° 8′ 56″ south and longitude: 38° 5′ 52” west). The pretreatment of the leaves was based on the methodology described by Ribeiro (2021) with modifications. After harvesting, the leaves were preselected and dried at 80 °C for 24 h in an (SL 102 model) oven with air circulation and renewal. Then, the leaves were powered in a knife mill and sieved into a 32-mesh granulometry (500 μm). The resulting powder was stored in properly closed polyethylene bags at room temperature (28 °C) and protected from light and heat.

### 2.2 Preparation and Characterization of Eutectic Mixtures

Hydrophilic eutectic solvents were prepared using choline chloride (Sigma-Aldrich, ≥98%) as the hydrogen bond acceptor (HBA) combined with various hydrogen bond donors (HBDs), including lactic acid (Dynamic, ≥85%), 1,2-propanediol (Sigma-Aldrich, ≥99%), and oxalic acid (Dynamic, ≥99.5%). A hydrophobic DES consisting of menthol as the HBA and lactic acid as the HBD was also produced. The DES preparations were performed following the methodology developed by Dai et al. (2013) for solid–liquid mixtures and Abbott et al. (2004) for solid–solid mixtures.^29,30^ The HBDs and HBAs were homogenized in baths at 60–80 °C for 30 min to achieve the eutectic point in the liquid phase.

All DESs were characterized for composition and structure by using Fourier transform infrared spectroscopy (FTIR) and nuclear magnetic resonance (NMR), respectively. The FTIR spectra were recorded using a Cary 630 FTIR spectrometer (Agilent Technologies) in the range of 650–4000 cm^–1^, with 32 scans and a resolution of 4 cm^–1^.^30,31,32^ Chemical analysis was performed using NMR (^1^H and ^13^C) of the samples, which were diluted in deuterated dimethyl sulfoxide (DMSO-*d*_6_) without pretreatment and placed in 5 mm diameter tubes. NMR experiments were performed by an NMR600-VNMRS600 spectrometer (600 MHz) at 25 °C, with 64 scans, 48k points in the time domain, a spectral window of 16.0 ppm, acquisition time of 5.0 s, and relaxation of 30.0 s.^33^ Chloride ion detection was carried out through volumetric quantification by titration with a AgNO_3_ solution (0.01 M) following the methodology described by Martins et al. (2016).^34^

The physicochemical characterization of the DES was performed by measuring pH using a PHS-3E pH meter (Satra) at 25 °C. Viscosity and density data were measured using an Anton Paar SVM 300 M viscometer, with temperatures ranging from 20 to 90 °C. Relative humidity was determined using the Karl Fischer method (Metrohm 870 KF Titrino Plus), with a 3:1 (v/v) chloroform/methanol solution. All analyses were performed in triplicate.^32^

### 2.3 Extraction Procedure

The extraction tests using DESs as solvents were conducted based on the methodology described by Ueda et al. and Santos et al. (2022) with modifications. Solid–liquid extractions were performed using two techniques: stirrer in a bath heated (SBH) and ultrasound bath technique (UBT). In the SBH method, extractions were carried out at temperatures ranging from 30 to 70 °C for 120 min, with a powder-to-DES ratio of 1:10 (m/v). For the UBT method, extractions were performed with the same component ratio and an operating time of 30 to 120 min at 30 °C. Comparative tests under the optimal operating conditions obtained from both methods were conducted using water and ethanol as conventional solvents. At the end of each extraction, the liquid phase was separated from the solid material by centrifugation (3500 rpm, 15 min) followed by vacuum filtration using a nylon filter with a pore size of 0.5 mm^2^.^35^ Both the extract and the remaining solid material were stored under refrigeration at – 18 °C.

Further extractions were performed using conventional methods and solvents. The Soxhlet method (SOX) was implemented in triplicate using a standard Soxhlet apparatus (250 mL) with leaf powder and methanol as the solvent in a ratio of 1/10 (m/v). This extraction was carried out over three cycles of 8 h each. Additionally, the SOX method was applied using ethanol (95%, Neom) as a solvent, at a ratio of 1/10 (m/v) to leaf powder, for 5 h in duplicate following the methodology described by Santos et al. (2022). The shaker in a thermostatic bath was also used with ethanol/water (50% v/v) as a solvent in a ratio of 1/20 (m/v). This extraction was carried out for 15 min at 50 °C, in duplicate, as described by Moraes et al. (2018).

### 2.4 Antioxidant Activity of the Extracts

The extracts were evaluated through assays to quantify the total antioxidant activity (TAA), a methodology adapted from Larrauri et al. (1997), and total phenolic compounds (TPCs), made following the methodology described by Obanda and Owor (1997). The readings were performed in triplicate by using acetate cuvettes and an Agilent Cary 300 UV–vis spectrophotometer. The TAA measurements were carried out at 734 nm using the ABTS^●+^ free radical capture method (2,2-azino-bis-(3-ethylbenzothiazoline-6-sulfonic acid) diammonium salt, Sigma-Aldrich, ≥98%) with Trolox (6-hydroxy-2,5,7,8-tetramethylchromane-2-carboxylic acid, Sigma-Aldrich, ≥97%) as the standard. The TPC measurements were performed at 700 nm using the Folin–Ciocalteu reagent (Scientific Exodus) with gallic acid (Dynamica, 98%) as the standard.

GraphPad Prism (version 9.00) was used to statistically analyze the antioxidant activities of the extracts. The results are presented as the standard deviation (±SD) of the mean and were evaluated by two-dimensional verification of variance (ANOVA) of pairs of means followed by the Tukey multiple comparisons test: between conditions of extraction to the same DES used and between the DES for each condition of extraction.

### 2.5 Scanning Electron Microscopy of Powder Leaves before and after Extraction

Morphological features of soursop leaf powder were examined by scanning electron microscopy (SEM). Before the SEM analysis, the samples were fixed on the stubs with carbon tape and then metalized with 20 nm of Au. The images were recorded by an FEI Quanta 450-FEG electron microscope at 1000–1500 kV. Images were generated with 100 μm (1300×), 10 μm (7500×), and 5 μm (15,000×) for each sample.

### 2.6 Chromatographic Analysis

The composition of the obtained extract in the exhaustive operation was determined by an ultra performance liquid chromatography system coupled to a mass spectrometer with a quadrupole analyzer and time of flight (UPLC/QTOF-MS, Waters, USA) following the procedures described by Costa et al. (2020). The UPLC procedure was operated with a Waters Acquity BEH C18 separation column (150 × 2.1 mm, 1.7 μm) set at 40 °C. An injection volume of an aliquot of 5 μL of the methanolic extract diluted (20 mg/mL) in acetonitrile (LiChrosolv, ≤30 ppm of H_2_O) and filtered with a hydrophilic PTFE filter (Analtica) with a pore diameter of 0.22 μm was used. The aliquot was subjected to an exploratory gradient of 30 min and a flow rate of 0.3 mL/min. The mobile phase consisted of deionized water and acetonitrile containing formic acid (0.1% v/v).^14,36^

## 3 Result and Discussion

### 3.1 Characterization of Total Extractives from Soursop Leaf Powder

Conventional solvents were used to extract bioactive compounds from soursop leaves to evaluate the total amount of extractives in more rigorous and prolonged processes. The methods employed are detailed in Table 1. The first method involves characterizing soursop leaf powder in terms of total extractive content by measuring the difference in dry mass before and after extraction using the exhaustive Soxhlet method with methanol as the solvent (boiling point of 64.7 °C). The results revealed a total extractive content of 24.31 ± 1.32% (SD) of the initial mass (before extraction), with a total antioxidant activity of 434.05 μM Trolox/g of dry mass and a total phenolic content of 107.99 mg of gallic acid/g of dry mass. These findings are consistent with other similar studies that also reported high values. These studies demonstrated that the pretreatment of the leaf powder and the Soxhlet extraction system do not degrade the bioactive compounds in the samples.^2,13^

**Table 1:** Characterization of Extractive Sample Soxhlet

analyses	Soxhlet methanol (1:10 m/v; 24 h)	Soxhlet ethanol (1:10 m/v; 5 h)	shaker ethanol 50% v/v (15 m; 50 °C; 1:20 m/v_solv._)
TAA^a^ μM Trolox/g, d.b.	434.05 ± 5.09	483.50 ± 3.01	419.75 ± 3.59
TPC^b^ μg GAE/g, d.b.	10,769.08 ± 406.09	12,728.92 ± 286.82	10,471.30 ± 1528.47

a Determined by the ABTS radical.

b Determined by the Folin–Ciocateu method.

To compare our results to those from other methods found in the literature, additional extractions were conducted. These included the Soxhlet extraction method using ethanol as the solvent for 5 h and the stirring method, which uses a mixer for only 15 min with a 50/50 v/v mixture of water and ethanol as the solvent. The total phenolic compound (TPC) results were consistent with those reported in reference studies, especially for the stirring method. However, the antioxidant activity values obtained in this study were lower than those in the references, and this difference may be attributed to several factors, including the inherent characteristics of the leaves (e.g., maturation stage and nutrient content) and the pretreatment processes applied to the biomass before extraction.^2,16^

The description of the bioactive compounds present in the Soxhlet/methanol extract was performed using UPLC chromatography. The chromatogram is shown in Figure S1 (Supporting Information), and the peak details are provided in Table 2. Several secondary metabolites were detected, including quinic acid (1.18 min), ferulic acid (4.7 min), rutin (5.75 min), and kaempferol (6.33 min), which are typical in plant matrix extractions. However, a group of molecules with a long carbon chain (C35) was also identified within the retention time range of 16.9 to 22.6 min. Several isomers of acetogenins, such as annonacin (C_35_H_64_O_7_), highlighted in Table 2, were identified. These isomers are characteristic of plants from the *Annona* genus and are bioactive compounds with high added value due to their potential in cancer cell treatment.^2,37−39^

**Table 2:** Identification of the UPLC Chromatogram Generated from the Methanolic Extract of the Soursop Leaf Powder

**peak no.**	**Rt min**	**[M-H]**^**–**^**observed**^a^	**[M-H]**^**–**^**calculated**	**product ions (MS/MS)**^b^	**molecular formula**	**ppm (error)**^c^	**putative name**^d^	**refs**
1	1.18	191.0547	191.0556	173	C_7_H_12_O_6_	–4.7	quinic acid^e^ (5)	(40,41)
2	3.06	315.0710	315.0716	152	C_13_H_16_O_9_	–1.9	unknown	(42)
3	4.18	163.039	163.0395	119	C_9_H_8_O_3_	–3.7	coumaric acid^e^	(40)
4	4.54	577.135	577.1346	425, 407, 289	C_30_H_26_O_12_	0.7	procyanidin B dimer^e^	(40,43)
5	4.76	193.0497	193.0501	179, 149	C_10_H_10_O_4_	–2.1	ferulic acid^e^	(40)
6	4.92	289.0702	289.0712	245, 203	C_15_H_14_O_6_	–3.5	catechin^e^	(40,43,44)
7	5.06	293.0872	293.0873	147, 165	C_11_H_18_O_9_	–0.3	dihydrojasmone	(45)
8	5.14	415.1225	415.1240	221, 239, 203	C_18_H_24_O_11_	–3.6	unknown	(45)
9	5.75	609.1437	609.1456	463, 301	C_27_H_30_O_16_	–3.1	rutin^e^	(42,43)
10	5.81	609.148	609.1456	463, 301	C_27_H_30_O_16_	3.4	rutin isomer	(42,43)
11	6.13	593.1495	593.1506	447, 285, 255	C_27_H_30_O_15_	1.9	kaempferol-*O-*hexosyl-*O-*rhamnoside isomer	(40,42)
12	6.33	593.1509	593.1506	447, 285, 255	C_27_H_30_O_15_	0.5	kaempferol-*O-*hexosyl-*O-*rhamnoside isomer	(42,43)
13	6.61	447.0912	447.0927	285, 255	C_21_H_20_O_11_	–3.4	kaempferol-*O*-hexoside	(42,46)
14	7.08	517.2274	517.2285	499, 487, 221, 205	C_24_H_38_O_12_	–2.1	unknown	(45)
15	16.09	277.2176	277.2168	253, 183, 112,	C_18_H_30_O_2_	2.9	unknown	(45)
16	16.45	277.2163	277.2168	253	C_18_H_30_O_2_	–1.8	unknown	(45)
17	18.29	611.4493	611.4523	575, 371, 353, 285	C_35_H_64_O_8_	–4.9	annopentocin C isomer	(45)
18	18.51	611.4520	611.4523	575, 371, 353, 285	C_35_H_64_O_8_	–0.5	annopentocin C	(45)
19	18.75	611.4545	611.4523	575, 371, 353, 285	C_35_H_64_O_8_	3.6	annopentocin C isomer	(45)
20	19.79	609.4355	609.4366	591, 439, 421, 437	C_35_H_62_O_8_	–1.8	annonisin	(45)
21	20.65	595.4562	595.4574	551, 483, 471, 343	C_35_H_64_O_7_	–2.0	annonacin isomer	(40,41,47)
22	21.37	595.4580	595.4574	551, 483, 471, 343	C_35_H_64_O_7_	1.0	annonacin isomer	(40,41,47)
**23**	**21.48**	**595.4577**	**595.4574**	**551, 483, 471, 343**	**C**_**35**_**H**_**64**_**O**_**7**_	**0.5**	**annonacin**	(40,41,47)
24	21.80	595.4585	595.4574	551, 483, 471, 343	C_35_H_64_O_7_	1.8	annonacin isomer	(40,41,47)

a Mass of the observed molecule minus one proton.

b Ion observed in the analysis.

c Mass error in ppm between the calculated and observed value.

d Compound nomenclature.

e Compared with the authentic standard.

The structure of annonacin is challenging to observe due to its long carbon chain, consisting of 35 to 37 carbon atoms, and the presence of γ-methyl and γ-lactone groups. These functional groups increase the susceptibility to cleavage, making structural analysis difficult.^38^ However, these same features enable the identification of potential molecular fragments in the presence of radicals, facilitating compound confirmation through comparison with its derivatives. Several studies in the literature provide strategies for identifying this sensitive compound using various chromatographic techniques.^37,40^ Based on these methods, the present investigation led to the conclusion that the compound belongs to the acetogenin class, confirming that the applied procedure effectively enabled its extraction. Figure S2 (Supporting Information) shows the TOF MS-ES mass spectrum and the derived radicals of peak 23, the most intense peak for an annonacin isomer.

### 3.2 DES Characterization

The produced DESs, their respective original substances, and their molar ratios are presented in Table 3. A total of five eutectic mixtures were produced, four of which were hydrophilic, using choline chloride as the HBA, and one hydrophobic, based on menthol. These eutectic mixtures were selected due to their successful application in previously reported extraction procedures involving different biomasses, including soursop leaves.^15,27,48−51^

**Table 3:**
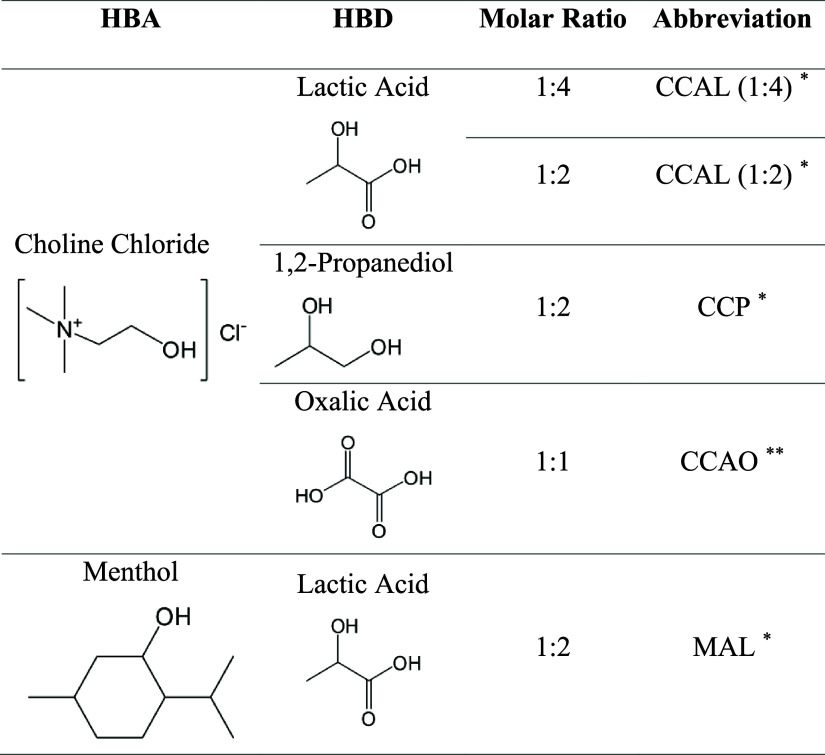
List and Composition of DESs Produced

a Produced according to the methodology proposed by Dai et al. (2013).

b Produced according to the methodology proposed by Abbott et al. (2004).

Specifically, CCAL (1:2) and CCAO were selected for their high efficiency in extracting alkaloids and phenolic compounds from leaves and fibers.^15,27^ Additionally, CCP had been previously applied for the extraction of bioactive compounds from soursop leaves by Santos et al. (2022). The primary objective was to compare the extraction efficiency of these acid-donor- and sugar-based DESs reported in the literature; however, two additional DESs were included: CCAL (1:4), to evaluate potential interferences associated with an increased concentration of the donor agent, and MAL, a hydrophobic DES. The use of a hydrophobic DES was also proposed by Ueda K. in his doctoral thesis, where it was briefly discussed, despite not being published, as a promising strategy for enhancing the extraction of phenolic compounds from uvaia leaves due to the chemical affinity. Considering that the extractives from soursop leaves are also hydrophobic polyketides, the inclusion of a hydrophobic DES could improve the stability of the extracted compounds and potentially facilitate their separation during the final processing stages.

Two DES production procedures were implemented due to the different physical states of the initial components. At first, the methodology described by Dai et al. (2013), which includes submitting the compounds to homogenization at 60 °C, was applied to the preparation of all DESs. However, modifications were necessary since not all combinations allow for the formation of DESs.

In this regard, for the CCAO production, the optimal molar ratio obtained is 1:1 (using dehydrated oxalic acid) at 80 °C, as predicted by other studies available in the literature.^17,52−54^ The 1:2 molar combination produced at 60 °C completely solidified at the end of the process. An indication of this behavior would be in the interactions with hydrogen bonds of the HBDs, which facilitate the development of parallel reactions, far away from the equilibrium condition, which is one of the characteristics of a eutectic point. Several authors have mentioned a tendency for solidification in DES systems where free water molecules are present, directly correlated with the hydration level of the initial components, increasing as the degree of hydration rises.^29,55,56^ In this case, the stable eutectic point for CCAO can be better obtained from a monohydrated HBD than from a dehydrated HBD.

A similar situation was figured out in CCP, which initially presented a homogeneous mixture at the end of the preparation at room temperature (25 °C) but after 24 h revealed needle-shaped colorless precipitates at the bottom of the container. This indicates that a 1:2 combination between choline chloride and 1,2-propanediol produces a certain imbalance that may become evident over time. The reason for the late appearance of the precipitate could be the adsorption of water to the DES mixture, which will be discussed later.^23,49,54^

**Figure 1:**
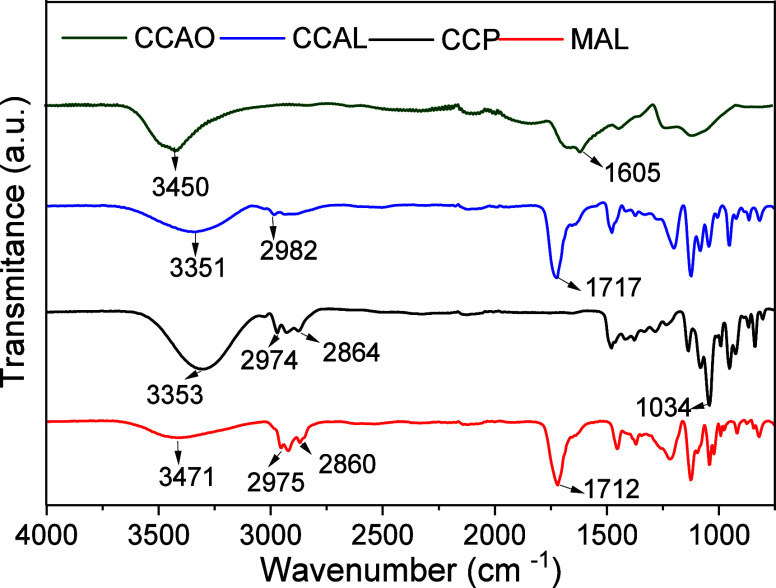
FTIR spectra of the produced DES.

Chemical information on the produced DESs can be determined through FTIR analysis (Figure 1). The FTIR patterns for DESs CCAL (1:2) and (1:4) are shown as CCAL in Figure 1 due to the same spectral pattern observed for both mixtures. The data obtained demonstrate that, for all DESs except CCP, the presence of the hydroxyl group represented by the wideband related to the O–H stretching vibration was detected in the region of 3351–3471 cm^–1^.^18,45^ CCP also shows an intense band at 3353 cm^–1^; however, the presence of a narrow and intense band at 1034 cm^–1^ refers to the C–N vibration and indicates a high concentration of unreacted choline chloride in the mixture, causing a molar imbalance in the system.^57^

The presence of two to three bands in the region of 2860–2982 cm^–1^ indicates stretching vibrations of aliphatic CH_2_ and CH_3_ groups in all DESs, except CCAO, where the carbons in the HBD structure are of the sp^2^ type.^22^ Additionally, in the region of 1712–1717 cm^–1^, both CCAL and MAL exhibit a band associated with the carbonyl group (C=O) of lactic acid.^22,23^ Finally, in CCAO, a short band observed at 1605 cm^–1^ may be related to the C=O bond of the −COO– cluster, which overlaps with the choline H–N cluster.^23^ These observations suggest that the HBD and HBA molecules coexist in the mixture medium of each eutectic system even after DES production. This indicates that the components are in equilibrium, providing the necessary conditions for DES formation.^21−23,58^

The NMR spectra of the DES confirm the composition of all of the formed mixtures, showing consistent patterns between the physical mixtures and the DES samples. This confirms that the original substances are still present in the final mixture, as expected for a DES. By definition, DESs require interactions between components to be exclusively covalent hydrogen bonding interactions, resulting in a thermodynamic mixture of the initial components rather than a new substance. The NMR results, shown in Figure S3 (Supporting Information), are consistent with those previously reported.^59−61^

The ^1^H NMR spectra for all DESs based on chloride choline have shown the signal in A-3.09 ppm (s, 9H) for N–N–N-trimethyl and the first triplet in B-3.39(t, 2H).^59^ However, signal C, which represents the triplet at 3.66–3.64 ppm to OH, is not observed in the CCP spectra, being overlapped by other signals of 1,2-propanediol. Regarding the ^13^C spectra, all DESs consisting of choline chloride exhibit the signals at 53.6 (A) and 55.5 ppm (B), but the third signal (D) is, as expected, obtained at 66.2 ppm in CCAL mixtures. Meanwhile, for CCP and CCAO, the signal is shifted to 67.4–66.6 ppm.^61^ Those differences may be related to the molecular interactions of the DES samples. Regarding CCP, the nonidentification of the C signal in ^1^H spectra indicates that the less sterically hindered COH group of 1,2-propanediol may be dissociated to release hydroxyls or form byproducts. As for the CCAO, the displacement means a greater electronegativity with an increase in the distance of the group from the TMS, resulting in a more acidic molecular condition for this DES.

For the spectra of the three DESs consisting of lactic acid, which are CCAL (1:4) and (1:2) and MAL, the presence of signals D-1.20 ppm (d, 3H) and E-4.01 ppm (q, 1H) is observed.^62^ The presence of the OH group was not identified, suggesting that this group may be interacting with hydrogen bonds, choline, or menthol. For the 1,2-propanediol molecule, the 1H spectra of CCP revealed the doublet at G-0.96 ppm (d,3H) and the H-3.80 ppm (m, 3H) and I-3.53 ppm (dd, 3H) signals overlapped with the choline signals. The ^13^C spectra to CCP showed no signal for the COH cluster of 1,2-propanediol and only exhibited signals for the other two carbons (G-20.4 and I-67.7 ppm). To CCAO, the spectra are more simplified due to the symmetry of the molecule, with only one signal appearing for hydrogens at J-4.57 ppm (s, 1H) and for carbons at J-161 ppm.^63^ The menthol molecule presents lots of interactions due to the presence of the ring, and most of these signals are shown to ^1^H since the K-0.69 ppm represents the singlet of 9H close to the methylene group to the duplet of 2H related to the CH_2_ in P-1.61 ppm. In the ^13^C spectra, the MAL signals from K-16.91 ppm to P-66.3 ppm and the more electronegative CH group of the ring appears in O-176.69 ppm.^64^

**Figure 2:**
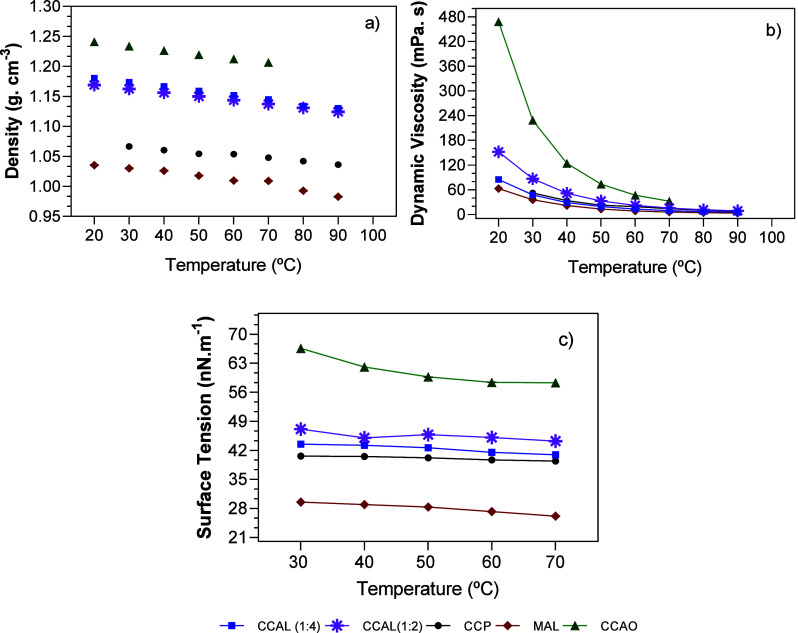
(a) Dynamic viscosity, (b) density, and (c) surface tension as a function of temperature for the produced DESs. Density was measured with an uncertainty of ±0.00005 g.cm^–3^, viscosity was measured with an uncertainty of ±0.35%, and surface tension was determined from 15 measurements per point, with an uncertainty of 0.01 mN·m^–1^, to a temperature variation of up to 0.1 °C measured externally.

At the end of production, all DESs presented a colorless mixture with a viscous appearance. Figure 2 shows the density and viscosities of each DES, where the pattern CCAO > CCAL (1:4) > CCAL (1:2)>CCP > MAL is observed for density and the pattern CCAO > CCAL (1:2) > CCAL (1:4) = CCP > MAL is obtained for dynamic viscosity and surface tension. As expected, the viscosity and density profiles show a decay with increasing temperature, and the slight variation in surface tension indicates high structural stability, even considering the weak interactions that govern the eutectic mixture. Even so, there were signs of degradation of the samples at temperatures above 90 °C and solidification at temperatures lower than 30 °C. To the CCAL samples, it can be noticed that the 1:4 combination is denser and at the same time less viscous than the 1:2 combination, with indicators that the high concentration of liquid HBD in the p system may favor the fluidity of the medium due to the excess of molecules available to interact with the HBA. Nonetheless, this same predisposition relatively increases the volume of molecules available per control volume, which would result in a small increase in viscosity.^48,55^ Thus, it can be suggested that eutectic mixtures are subject to intermolecular interactions, along with isenthalpic and isentropic processes such as volume, shape, and molecular size.

The CCAO has the highest viscosity and the highest density at all temperatures, as expected due to the donor agent having two available hydroxyl groups, which allow for multiple ionic interactions with the choline molecules, even a pairing of interconnected molecules, leading to a very dense formation of the mixture.^48,55^ Meanwhile, MAL has the lowest viscosity and density values compared to the other three, likely due to hydrogen bonding that leads to the formation of this DES. In MAL, the interaction with the lactic acid hydroxyl occurs with the hydroxyl available in the menthol molecule, characterizing a weaker connection than that in choline-based DES, in which the receptor is a strongly electronegative chloride ion. Despite these indications, explanations for this behavior need to be elucidated.

**Figure 3:**
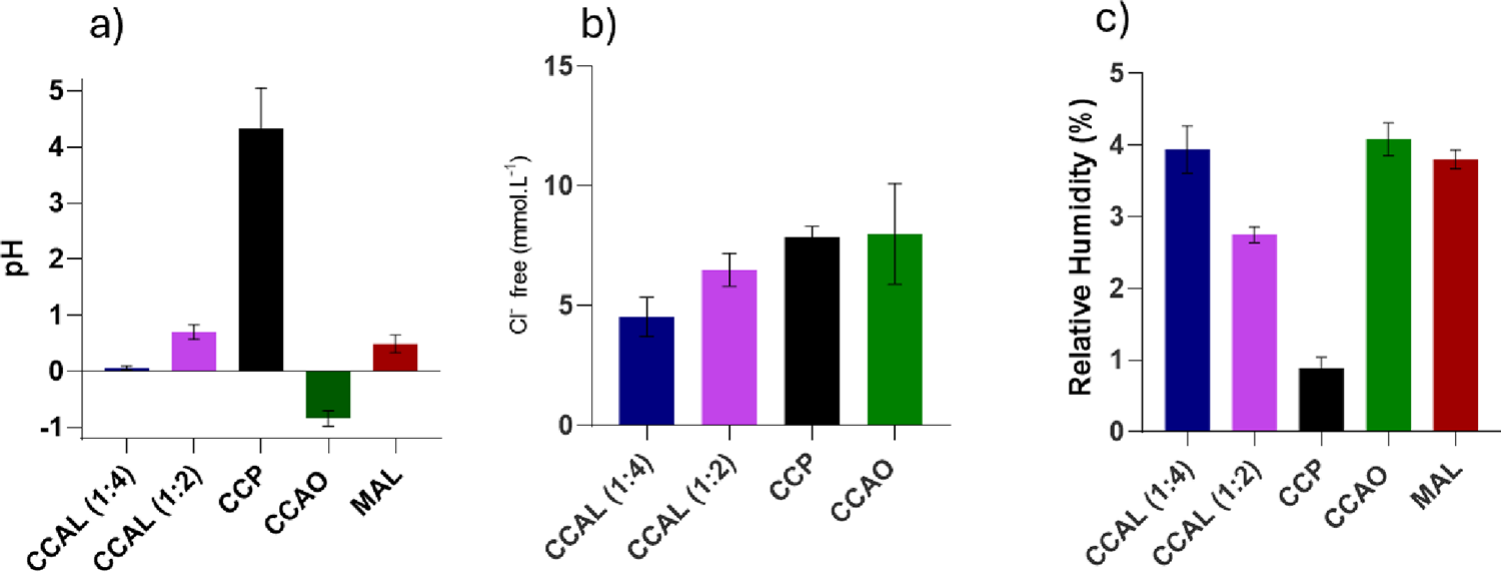
Physicochemical characterization of DES: (a) pH of eutectic mixtures at 25 °C. (b) Concentration of Cl^–^ by AgNO_3_ titration. (c) Relative humidity of all DESs.

Regarding the pH analysis, it is evident that all DESs (deep eutectic solvents) exhibit acidity, as shown in Figure 3. These mixtures possess highly corrosive properties, which cannot be solely attributed to the hydrogen-accepting agent (choline, with a pH of 6.5 at 25 °C) or the hydrogen-donating agent. This is evident because the precursor compounds of these mixtures do not exhibit a similar level of acidity or proximity to it. For instance, lactic acid, which is present in CCAL and MAL, has a pH equal to 5.5 at 25 °C, and in the same way, 1,2-propanediol, at 25 °C, has a pH of around 7. The most acidic component is oxalic acid, with a pH of around 1.5 at 25 °C, but it would not justify such a low pH in CCAO. This acidity may be due to the free chloride ions, which could interact with H molecules in the medium to form HCl. To prove this hypothesis, a titrimetric test was performed with AgNO_3_ to verify the chloride ions. The formation of a milky mixture was observed in all tests, indicating the presence of Cl^–^ ions. The formation of HCl in the medium would then be entirely possible, and given that pure HCl has a pH of −1.1 at 25 °C, its presence would be responsible for the decrease in the pH of the mixture.^65,66^

The relative humidity data for DESs are also presented in Figure 3. It is generally observed that the humidity remains below 5% even for hydrophilic DESs, indicating the stability of the mixtures against water absorption from the air. In this case, the high hydrophilicity of pure HBD does not interfere with the formation of the eutectic point, indicating an interaction between HBD and HBA in the medium. Among all of the samples, CCPR has the lowest moisture percentage (1%), corroborating the premise that precipitation occurs due to interaction with the maximum quantity of hydroxyls available in the medium. This decreases the amount of water molecules available in the mixture and evidences the formation of a precipitate, as it was visually observed after 24 h.

The other hydrophilic DESs show low moisture due to their compositions. For CCAO, the arrangement of a single HBD molecule per HBA in the medium drastically reduces the possibility of parallel interactions and water release, as indicated by the density and viscosity results. Finally, the DES considered hydrophobic, MAL, has the second-highest relative humidity, indicating that the solubility of DESs barely supports a certain amount of water. Consequently, this solubility can improve the motility of the molecules and reduce the density and viscosity of DESs, as previously observed. Characterizations of DESs offer a justification for their performance in the extraction tests and help justify the extracted components, as presented in the following topic.

### 3.3 Bioactive Compound Extraction

The DESs were tested in a sorting system using the SBH method based on the reference information provided by Ueda et al. (2022) and Leal et al. (2022). However, unexpected observations occurred during the extraction operation, such as the odor released from the extraction system varying with the temperature changes. Initially, for the tests at 30 °C, the natural smell of soursop leaf was observed, but in the tests with CCAO and CCP at 50 and 70 °C, a fish-like odor associated with the characteristic odor of choline was noted after 30 min. In the extraction with MAL, menthol’s characteristic odor was noted, which became pungent at 70 °C. This may be due to the possible degradation and volatilization of DESs at temperatures above 60 °C, as observed in other works available in the literature.^17,48^ Additionally, the mixtures, which initially presented the same greenish hue of leaf powder, became dark brown for all DESs after 120 min of extraction.

**Figure 4:**
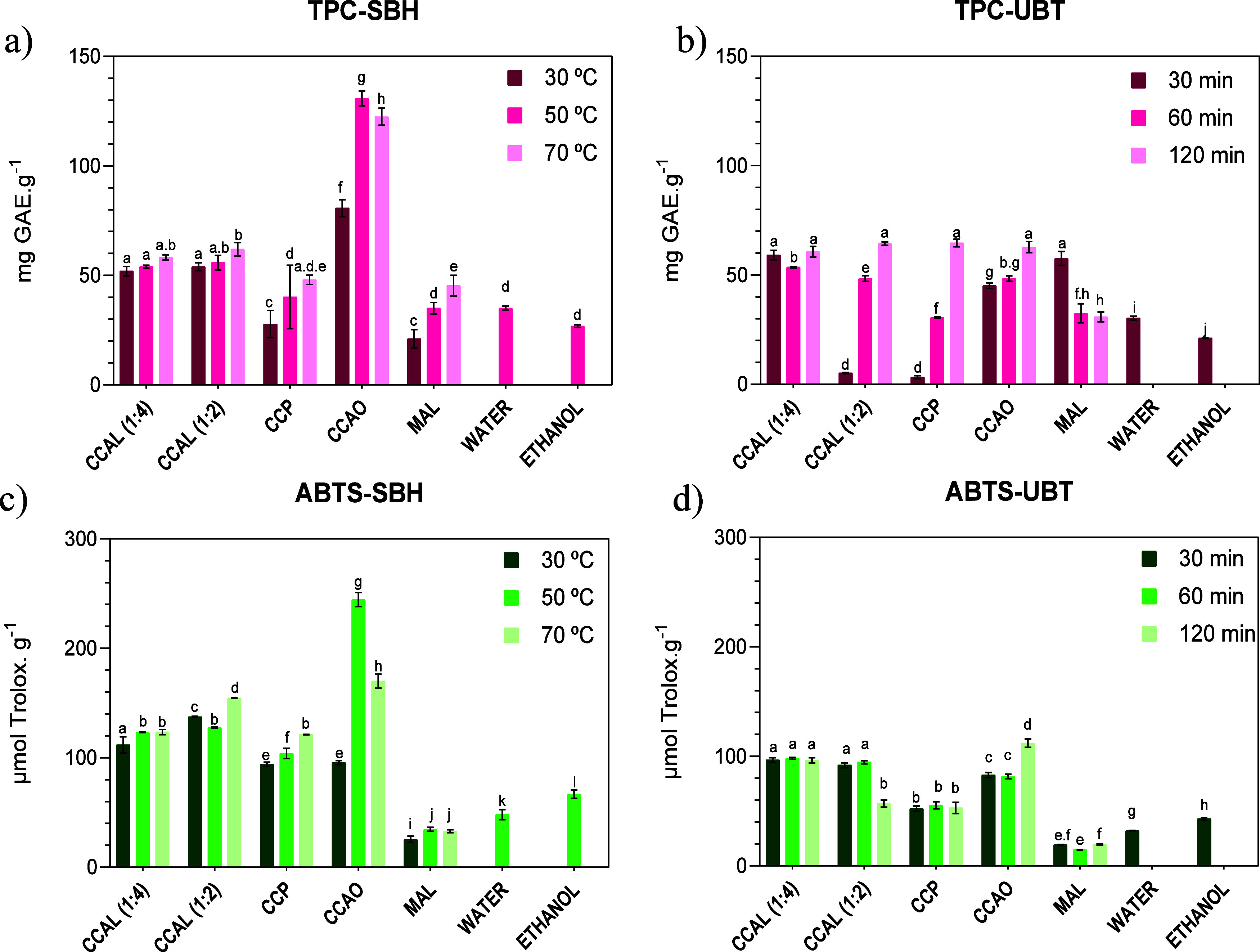
Experimental values of the group of extractives obtained by DES extraction: total phenolic compounds to (a) SBH and (b) UBT methods, and total antioxidant activity of the ABTS radical to (c) SBH and (d) UBT extraction. Values are the mean ± SD (*n* ≥ 2). Statistical analysis was performed by two-way ANOVA using Tukey’s multiple comparisons test. The same letters represent no significant differences at the 95% confidence level.

The results of the antioxidant activity and total phenolics are presented in Figure 4. It can be observed for SBH extractions that increasing the temperature leads to a rise in the number of antioxidants and phenolic compounds. However, this observation does not show enough significant cost–benefit implications, as is evident in the CCAL results. Additionally, it does not justify the higher energy expenditures to obtain a marginal increase in extractives, as indicated by the ANOVA probabilistic test. Therefore, 50 °C would be sufficient to obtain the desired extractives and is considered the optimal point for SBH in the CCAO extraction method.

Due to the high viscosity of DESs, the diffusivity and solubility are the most cited reasons to explain the performance of extractions at higher temperatures. This condition may be the reason why the results of extractions at 30 °C are slightly lower than those at 50 and 70 °C, but the high acidity of the materials has already been pointed out in other investigations as the main reason to promote extractions.^17,19,48^ In this regard, CCAO stands out as the solvent with the highest capacity to extract bioactive compounds, with total antioxidant activity (244.38 mmol Trolox/g of the sample at 50 °C) and total phenolics (130.89 mg GAE/g of the sample at 50 °C) in the SBH method that are higher than all other tests. It is worth emphasizing that the CCAO, even if it is the densest and most viscous mixture, can promote high extraction performance in both the SBH and UBT methods, and this is due to the high acid conditions obtained from the HBD and HBA interaction.

The extraction with CCAO promotes greater exposure of intracellular compounds and higher extraction efficiency due to the breakdown of fiber structural components, as previously reported for the extraction of lignin and cellulose from sugar cane.^67^ This effect will be confirmed by the SEM analysis of the biomass surface latter. The acidic nature of CCAO facilitates the protonation of certain functional groups in the target compounds, like rutin, quercetin, and anthocyanins, improving their solubility in the DES.^17,29^ For phenolic acid compounds like quinic coumaric and ferulic acids, the extraction in acidic environment increases their solubility and stability, preventing oxidation.^29,68,69^

The results obtained from CCAL show no difference between the 1:4 and 1:2 composition to the TPC and ABTS tests, considering the same temperature of the samples. This sample also showed little variation in the increase in temperature, indicating a very stable mixture. In intermediate positions on the extraction, there is CCP that, even with the precipitated material, was still able to promote extractions. In this case, all hydroxyl groups are suppressed, keeping them connected and active in hydrogen bonds in the medium. This condition reduces the possibility of interactions that could lead to the formation of water and, subsequently, the dissociation to produce HCl, detected as a decrease in moisture and an increase in pH. All of these interactions were probably the reason why CCP did not stand out as a good solvent extractor.

Finally, the extractions with MAL showed the lowest results of bioactive extraction due to the apparent degradation and volatilization of the solvent. Even with lower density and viscosity, this mixture cannot perform better in the extraction process. This implies that solid–liquid extractions with DES are driven by physicochemical properties and isotropic and isotropic processes that need to be significantly understood to improve and optimize the process.

The resulting antioxidant activity obtained from the ultrasonic bath test, shown in Figure 4, is less polarized than that achieved with the SBH method. Among the solvents tested with SBH, CCAO demonstrated the highest activity, allowing extractions of up to 112.10 μM Trolox/g of sample in 120 h of operation. This result is similar to the TPC results obtained with CCAL at 1:4 and 1:2 ratios, as well as with the MAL method. Considering the effectiveness of extractions, the ideal time for the operation would be 30 min. Longer times would promote a decrease in the amount of antioxidant material available. Thus, it is likely that some molecular degradation occurred due to the time of exposure to ultrasound. Extractions with MAL were again the least efficient, indicating that the hydrophobic conditions of this mixture are not ideal for this extraction system.

Unexpected differences were observed in the analyses of total phenolics in the UBT method, where an excess of activity was observed for the DES MAL, which had been identified as the least active compared with the other tests. The chemical characteristics of DES, however, favor different affinities with the bioactive compounds in the system in such a way that DES can be designed for the exclusive extraction of a certain group of extractives to the detriment of others, as pointed out by Ueda et al. (2022).

The UBT method was less effective than the SBH on the extractions by not promoting any form of heating or macroscale agitation of the system. Nevertheless, it was possible to identify the differential effect of the physical–chemical properties of the system in the solid–liquid extraction method with DES. Heating and agitation processes can favor most biomass extraction methods by promoting more vigorous plant cell disruption. However, the chemical composition of DES and its effect on matrix interaction may be a second pathway that supports the extraction method.

Comparing with the literature, Santos et al. (2022) used CCP in UBT to extract compounds from soursop leaves, achieving 6 mg GAE/g after 30 min, while this study reported 3.26 mg GAE/g. However, for longer extraction times, this study showed higher results, reaching 64 mg GAE/g in 2 h compared to 45 mg GAE/g after 3 h in the literature. The previous study included vigorous agitation and a solid-to-liquid ratio of 1:20, which likely improved extraction efficiency. However, it did not mention the presence of a precipitate in CCP, which may have contributed to the higher efficiency observed in this study due to more acidic conditions.

The extractives obtained with the different DESs showed consistent results in terms of the major compounds identified by ABTS and TPC, particularly for CCAL (1:2) and CCP (1:4), as well as for CCP and MAL extractions. Significant differences were observed only for the most acidic DES, CCAO, which presented the highest results. These variations are consistent with previous studies that highlight the influence of DES composition on extraction efficiency and selectivity.^16,17^ The antioxidant activity data also indicate consistency in values, considering the effect of temperature and operation time in the different methods applied. Gradual increases or slight degradations were observed at higher temperatures, as expected. For the same DES, slight variations related to temperature changes, even across different extraction methods, were also verified, indicating operational consistency.

It is important to mention that some extractions were performed in duplicate and analyzed to evaluate the replicability of the test, including the unexpected result observed for CCAL 1:2 and CCP extractions in the UBT method at 30 min. No significant differences were observed between the results aside from the expected experimental error associated with the spectrophotometric procedure.

To compare with conventional methods, extractions were also performed using ethanol and water as solvents under the optimal conditions observed in the SBH and UBT methods. It was noted that ethanol and water may not be as efficient as DES, as observed in other studies. Thus, in addition to being considered favorable given its benefits in terms of biodegradability and environmental disposal, DES is also advantageous for promoting greater extraction efficiency, as already discussed.

**Figure 5:**
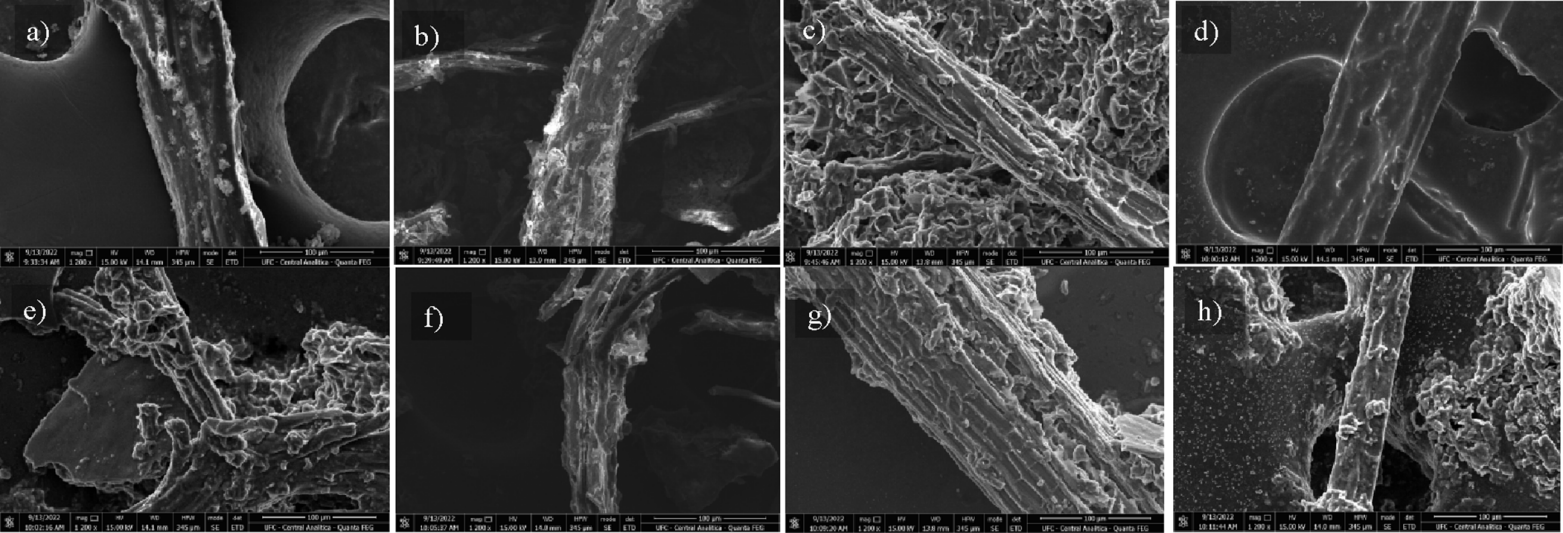
SEM of *Annona muricata* powder samples. (a) Before the extraction. After extraction: (b) SOX methanol of 24 h, (c) SBH with CCAL 1:4, (d) SBH with CCAO, (e) SBH CCP, (f) SBH with MAL, (g) UBT CCAL 1:4, and (h) UBT CCP.

The biomass residue remaining after each extraction procedure was analyzed through morphological assessments. A scanning electron microscope was used to evaluate potential topographical changes in the leaf powder samples before and after extraction, with the results presented in Figure 5.

The image of the sample without any extraction treatment, shown in Figure 5a, presents a smooth surface with scattered and adhered particle aggregates, likely from remnants of the leaf grinding process, although the fiber did not show any scratches. After the extraction process, several changes can be identified, some more pronounced than others, such as in the case of the Soxhlet extraction method (Figure 5b), which produced fibers with a surface similar to that of the untreated fibers, with slight variations in particle aggregation.

The images of fibers treated with CCAL1:4 (Figure 5c) and CCAO (Figure 5d) indicate that these DESs, particularly CCAO, provide more significant rupture and shear on the biomass surface due to their strongly acidic conditions. The high acidity enhances the degradation of the biomass surface, leading to increased disruption of the cell wall matrix. This corroborates the results of TAA and TPC, which were more expressive than others. On the other hand, exhaustive methods such as SOX, even considering a long time of operation, did not promote topographical differences in the material.

Compared to the initial sample, the extractions with MAL showed in Figure 5f resulted in a surface of soursop fibers similar to that before extraction. This indicates that this procedure was not efficient for the treatment of biomass, which requires greater friction or high acid conditions to release the extractives. This also corroborates the results of poor antioxidant activity obtained for the extractions with this DES. On the other hand, the samples treated with CCAL and CCP (Figure 5g,h) by the UBT procedure exhibited a significant number of fissures, indicating pronounced shearing. The samples treated with CCAL by the UBT method presented greater surface roughness in the fibers, while the sample treated with CCP presented dotted particles throughout the surface, suggesting the presence of residual solid material.

The results corroborate the premise that DESs with more acidity can actively participate in the extraction process, differing from conventional solvents that strongly depend on operating conditions, such as vigorous agitation and heating, to ensure diffusion processes of the molecules of interest from inside plant cells to the extractive medium. The advantage of using DESs in extraction systems lies in the fact that DESs provide physicochemical conditions to break the plant’s cell wall. This active action of the solvents can be useful to the point of facilitating the extraction, allowing for the use of milder systems in operations with plant matrices.

## 4 Conclusions

This study evaluated the application of different types of deep eutectic solvents (DESs) on the extraction efficiency of bioactive compounds *Annona muricata L.* leaves under optimal operating conditions, including temperature, solvent-to-biomass ratio, and extraction time, in a solid–liquid system. Additionally, it compared conventional extraction techniques, such as mechanical agitation and ultrasonic bath, to identify the most efficient method in the presence of DESs. Bioactive compound extraction from soursop leaves was performed with DESs, showing an optimal reaction point at 50 °C, with a ratio of 1:10 m/v between the biomass and the solvent volume. Compared with conventional extraction methods, the DESs studied demonstrated greater efficiency and stability, facilitating plant cell wall disruption and promoting more effective extraction. The CCAO DES exhibited the best extraction performance due to its acidic properties, despite its high viscosity and density. Furthermore, all DESs showed high acidity, a distinctive characteristic that contributed to enhanced extractions. This study significantly contributes to the field of green chemistry by demonstrating that DESs are a sustainable and efficient alternative for extracting bioactive compounds. The findings pave the way for future research and industrial applications, offering an innovative and environmentally friendly approach for extracting high-value biocompounds from plant biomass.

## Supporting Information Available

Chromatogram QTOF MS-ES of the methanolic extract of soursop leaf powder obtained in the Soxhlet system and TOF MS-ES mass spectrum; ^1^H and ^13^C NMR spectra for all DESs produced and molecules identified for each DES structure (PDF)
