# Supplementary material for: Sustainable Extraction of Bioactive Compounds from Annona muricata L. Leaves by Deep Eutectic Solvents (DESs)
Source: ACS Omega. 2025 Apr 2;10(16):16909–20. doi: 10.1021/acsomega.5c01232 (PMC12044472; doi:10.1021/acsomega.5c01232)
Supplement: Supplementary file 1 — ao5c01232_si_001.pdf [file ao5c01232_si_001.pdf]

SUPPORTING INFORMATION

SUSTAINABLE EXTRACTION OF BIOACTIVE COMPOUNDS FROM *Annona muricata L.* LEAVES BY DEEP EUTECTIC SOLVENTS (DES)

Lais F. Oton†, Paulo R. V. Ribeiro‡, Edy S. de Brito ‡, Rílvia S. de Santiago-Aguiar †*

† Chemical Engineering Department, Federal University of Ceará, Pici Campus, Bloco 731B, 60440-900, Fortaleza, CE, Brazil

‡ Embrapa Tropical Agroindustry, Rua Dra Sara Mesquita 2270, Planalto do Pici, CEP 60511-110 Fortaleza, CE, Brazil

***CorrespondingAuthor:** Rílvia Saraiva de Santiago-Aguiar

Department of Chemical Engineering

Federal University of Ceara

Campus do Pici, Bl 709, Fortaleza, CE

Zip code: 60440-900

phone: +55 85 3366-9611; fax: +55 85 3366-9610

E-mail: rilvia@ufc.br

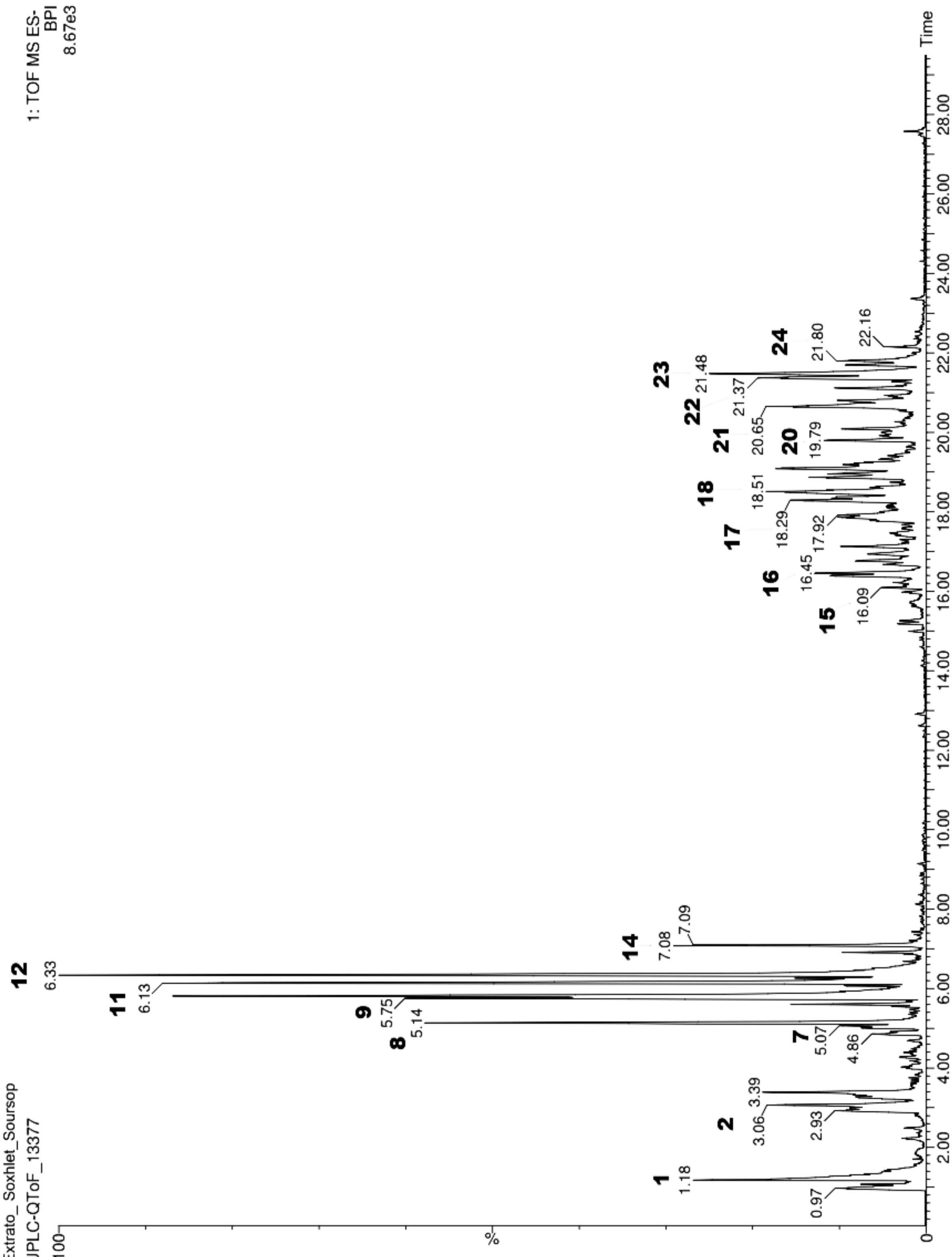

Figure S1. Chromatogram QTOF MS-ES of methanolic extract of soursop leaf powder obtained in Soxhlet system.

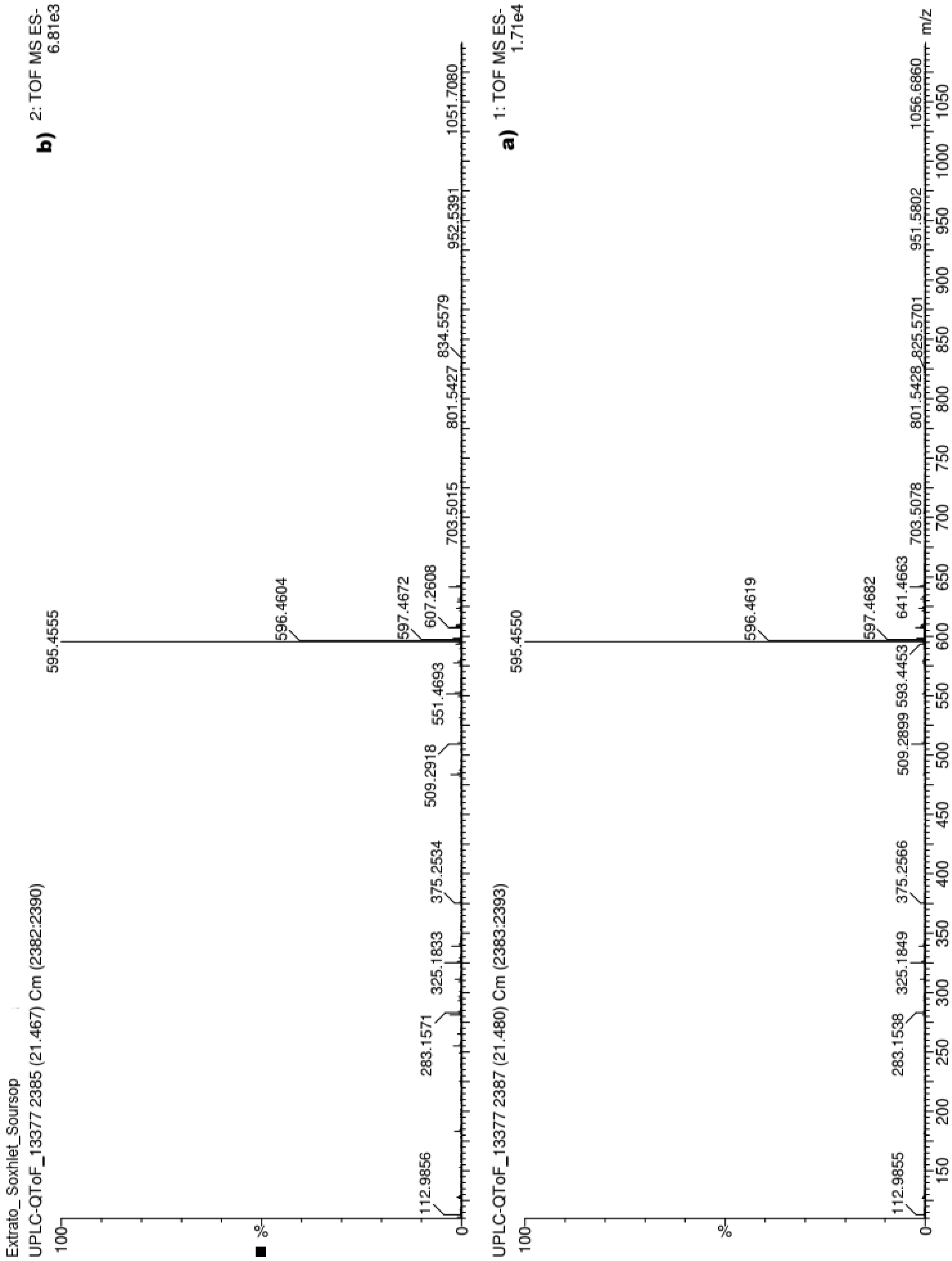

Figure S2. TOF MS-ES mass spectrum a) for the peak detected at 21.467 min and b) its derived radicals.

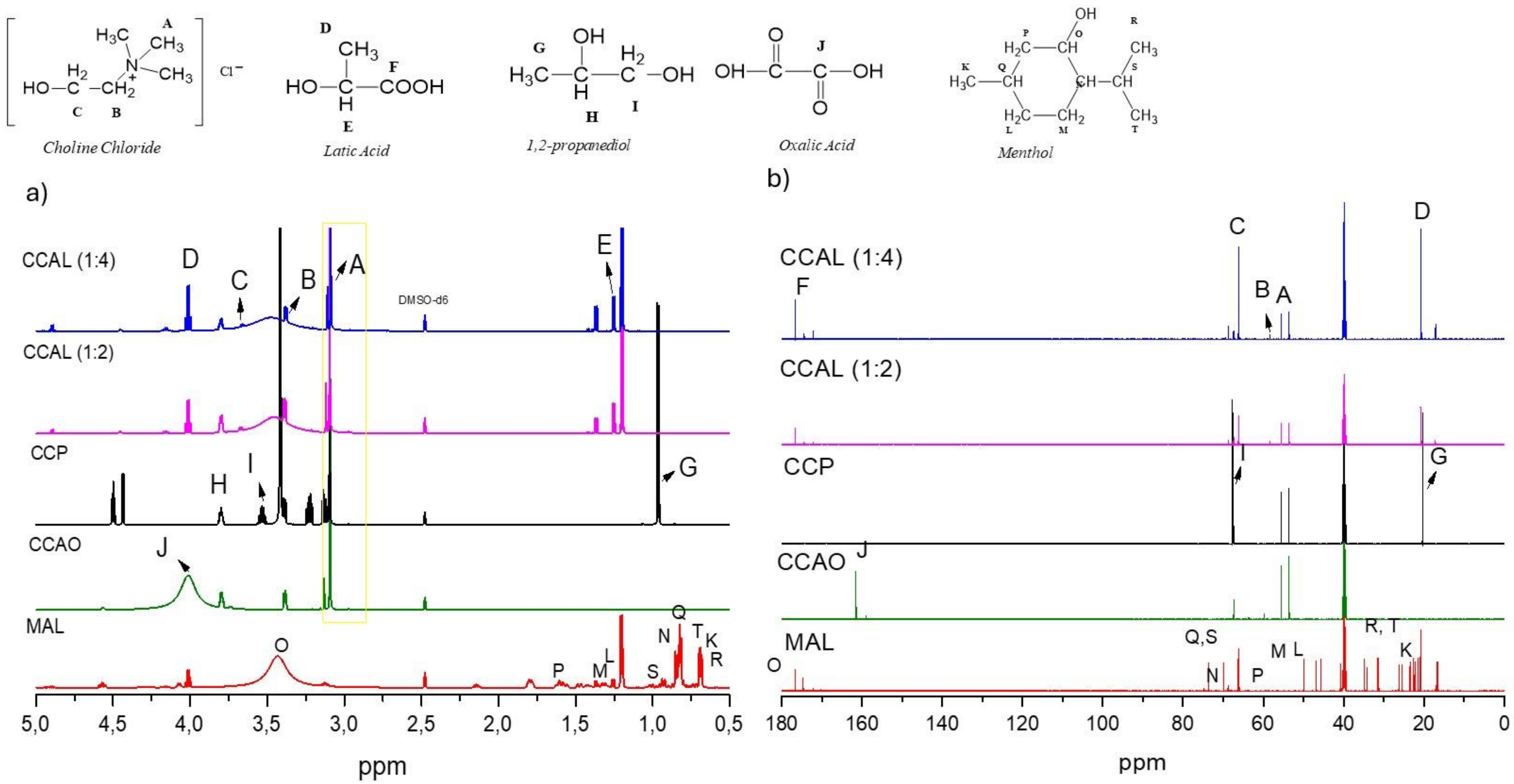

Figure S3. NMR patterns for all DES produced and molecules identified for each DES structure: a) ^{1}H NMR b) ^{13}C NMR
